# Serum and mucosal antibody-mediated protection and identification of asymptomatic respiratory syncytial virus infection in community-dwelling older adults in Europe

**DOI:** 10.3389/fimmu.2024.1448578

**Published:** 2024-10-18

**Authors:** Deniz Öner, Charlotte Vernhes, Sunita Balla-Jhagjhoorsingh, Annick Moureau, Marjolein Crabbe, Bruno Salaun, Arangassery Rosemary Bastian, Kim Thys, Jonathan De Smedt, Salo N. Ooft, Koos Korsten, Niels Adriaenssens, Samuel Coenen, Christopher C. Butler, Theo J. M. Verheij, Simon B. Drysdale, Joanne G. Wildenbeest, Andrew J. Pollard, Peter J. M. Openshaw, Louis Bont, Jeroen Aerssens

**Affiliations:** ^1^ Translational Biomarkers Infectious Diseases & Statistics, Janssen Research & Development, Beerse, Belgium; ^2^ Vaccines R&D, Sanofi, Lyon, France; ^3^ Clinical Immunology, Janssen Vaccines and Prevention BV, Leiden, Netherlands; ^4^ Vaccines R&D, GlaxoSmithKline (GSK), Rixensart, Belgium; ^5^ Department of Paediatric Infectious Diseases and Immunology, Wilhelmina Children’s Hospital, University Medical Center Utrecht, Utrecht, Netherlands; ^6^ Department of Medical Microbiology and Infection Prevention, Amsterdam University Medical Centre, Amsterdam University, Amsterdam, Netherlands; ^7^ Department of Primary and Interdisciplinary Care (ELIZA)-Centre for General Practice, University of Antwerp, Faculty of Medicine and Health Sciences, Antwerp, Belgium; ^8^ Laboratory of Medical Microbiology, Vaccine and Infectious Disease Institute (VAXINFECTIO), University of Antwerp, Antwerp, Belgium; ^9^ Nuffield Department of Primary Care Health Sciences, University of Oxford, Oxford, United Kingdom; ^10^ Julius Center for Health Sciences and Primary Care, University Medical Center Utrecht, Utrecht, Netherlands; ^11^ Oxford Vaccine Group, Department of Paediatrics, University of Oxford, and the National Institute for Health and Care Research (NIHR) Oxford Biomedical Research Centre, Oxford, United Kingdom; ^12^ Centre for Neonatal and Paediatric Infection, Institute for Infection and Immunity, St George’s, University of London, London, United Kingdom; ^13^ National Heart and Lung Institute, Imperial College London, London, United Kingdom

**Keywords:** respiratory syncytial virus, RSV infections, older adults, immune correlates, immune response, symptomatic infections, humoral immunity

## Abstract

**Introduction:**

Respiratory syncytial virus (RSV) causes acute respiratory tract infection (ARTI) and reinfects adults throughout life, posing a risk for hospitalization in older adults (>60 years) with frailty and comorbidities.

**Methods:**

To investigate serum and mucosal antibodies for protection against RSV infections, baseline serum samples were compared for RSV-pre- and -post-fusion (F) binding, and RSV-A2 neutralizing IgG antibodies between symptomatic RSV-ARTI (*N* = 30), non-RSV (RSV negative) ARTI (*N* = 386), and no ARTI (*N* = 338). Mucosal RSV-pre-F IgA and IgG levels, as well as serum RSV-G IgG antibodies, were analyzed to determine their association with protection from symptomatic RSV-ARTI in a subset study.

**Results:**

Using a receiver operating characteristic (ROC) analysis, we established thresholds of 1.4- to 1.6-fold change (FC) for RSV-pre-F and -post-F, and RSV-A2 neutralizing IgG antibodies, respectively, enabling the identification of asymptomatic RSV cases with high sensitivity and specificity (>80% and >90%, respectively). As a result, serum RSV-pre-F, RSV-G IgG, and mucosal pre-F binding IgA antibodies showed correlations with protection against symptomatic RSV infection. RSV-pre-F IgG antibodies were correlated with protection from RSV infections irrespective of the symptoms.

**Discussion:**

This study provides insights into antibody-mediated protection for symptomatic RSV infection in a community-dwelling older-adult population and establishes a threshold to identify asymptomatic RSV infection using a data-driven approach.

## 1 Introduction

Respiratory syncytial virus (RSV) is a prevalent pathogen causing acute respiratory tract infections (ARTI) and hospitalizations among older adults (>60 years old) with frailties and comorbidities (1–3). In industrialized countries, 1.5 million episodes of RSV-ARTI in older adults are estimated, and of these, approximately 14.5% resulted in hospital admission (4). In our longitudinal study conducted across three European countries [from the Respiratory Syncytial Virus Consortium in Europe (RESCEU)], we found that 4.2% and 7.2% of community-dwelling adults over 60 years old were infected with RSV during two consecutive seasons (2017–2019). Among those infected, one-third sought medical attention, but none needed to be hospitalized (5).

Previous studies have reported the role of serum and/or mucosal antibodies in protecting older adults from RSV infection (6). Lower levels of RSV neutralizing antibodies were associated with a higher risk of RSV infection in adults with community-acquired pneumonia (7), in frail older adults (8), in a population including healthy young adults, in community-dwelling older adults, in community-dwelling adults with comorbidities (9), and in a human challenge study (10, 11). Lower levels of IgA to RSV in the nasal mucosa have also been associated with an increased risk of RSV infection in a human challenge study in younger adults (11, 12) and in an adult population including healthy young adults and older adults with and without comorbidities (9). However, to our knowledge, no research has simultaneously evaluated antibody-mediated protection in community-dwelling older adults in Europe, considering both nasal mucosa and serum samples.

To address this gap, we utilized the RESCEU older-adult cohort to investigate serum and mucosal antibody-mediated protection, by comparing participants with symptomatic RSV ARTI with those with no symptomatic ARTI (no ARTI), non-RSV ARTI, and asymptomatic RSV, the latter identified with a data-driven machine learning approach. We found a correlation between serum pre-fusion (F) binding IgG, serum G binding antibodies, and mucosal pre-F binding IgA antibodies and protection from symptomatic infection. This work not only provides an understanding of antibody-mediated protection for RSV disease in older adults, but also establishes a threshold to detect asymptomatic RSV infection in older adults.

## 2 Methods

### 2.1 RESCEU older-adult cohort study design

The RESCEU older-adult study design, study population, and identification of respiratory infections have been described previously (5). In brief, the RESCEU older adult study is a multi-country, multi-center, longitudinal, prospective, and observational cohort study. It is the largest epidemiological study within Europe that aimed to estimate the burden of RSV disease (Clinicaltrials.gov, identifier: NCT03621930).

**Figure 1:**
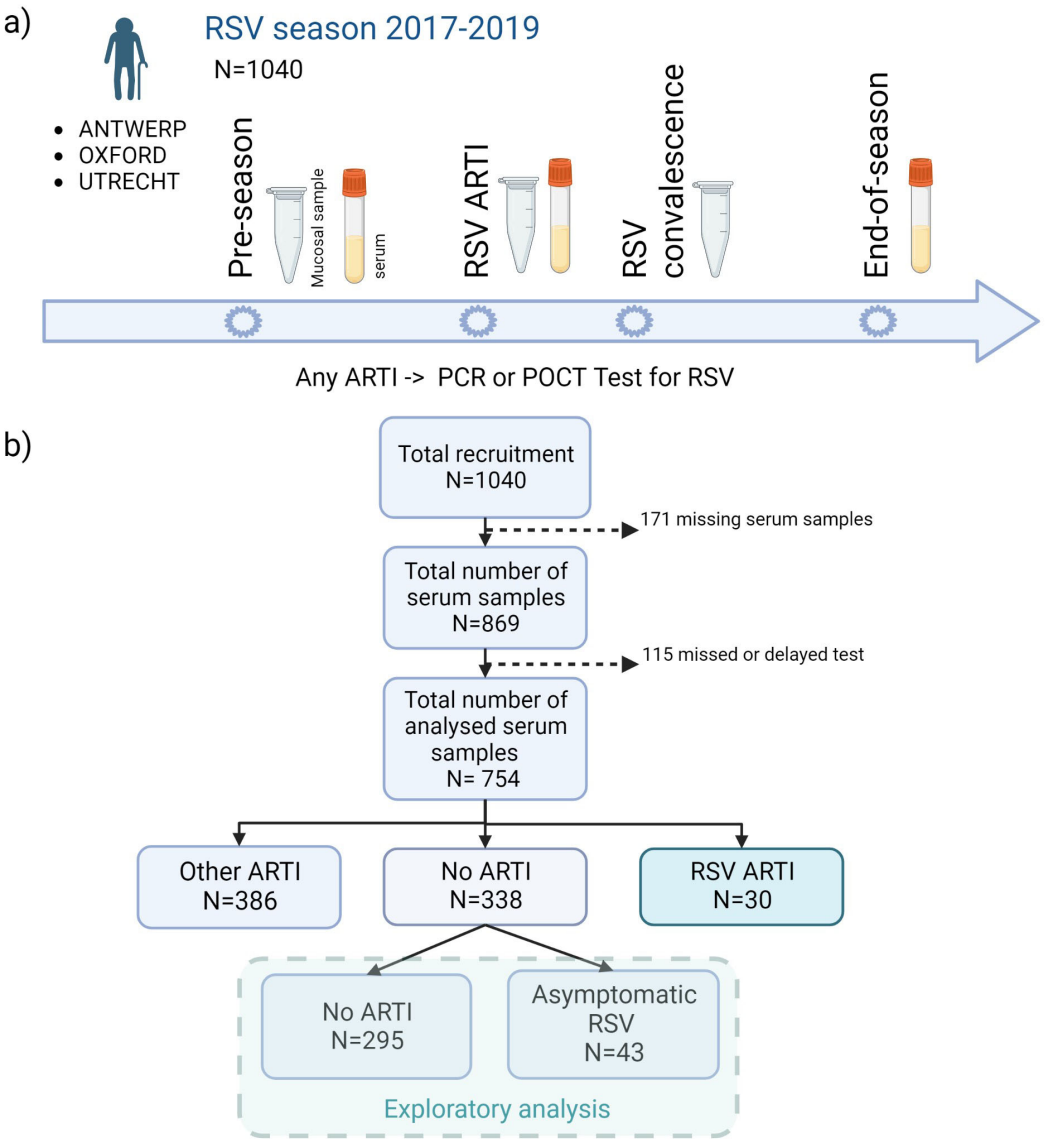
Flowchart of the study design. **(A)** A total of 1,040 older adults were recruited before the start of two consecutive (2017–2019) RSV seasons (1 October to 1 May) and followed up during one season. In case of any ARTI, an RSV POCT RSV test was performed (results confirmed by qPCR). Mucosal (i.e., nasal swabs) and/or serum samples were collected at pre-RSV-season, RSV ARTI, and end-of-season visits. **(B)** After the exclusion of missing samples and participants with missed or delayed test, pre-RSV-season antibody levels of 754 participants were investigated for (symptomatic and asymptomatic) RSV ARTI compared with controls. ARTI, acute respiratory tract infection; N, number of participants; POCT, point-of-care test; PCR, polymerase chain reaction. Created with Biorender.com.

Overall, 1,040 adults older than 60 years of age were recruited from the general population before the start of two consecutive (2017–2019) RSV seasons (1 October to 1 May of the subsequent year) and followed up during the course of one season. The individuals were recruited from the general community in the Netherlands (University Medical Centre Utrecht), Belgium (University of Antwerp), and United Kingdom (Oxford University Hospitals). The presence of respiratory symptoms triggered a study visit and sample collection (mucosal and/or serum), after which participants kept a symptom diary for the course of their illness (5) (**Figure 1A**). Participants with ARTI symptoms were tested using molecular tests, and seroconversion was determined by comparing end-of-RSV-season to pre-RSV-season antibody levels.

### 2.2 Study sample collection timeline

Serum samples were collected at pre-RSV season, RSV ARTI, and end-of-RSV season visits. Nasopharyngeal swabs (mucosal samples) were collected at pre-RSV season, RSV ARTI, and RSV convalescence. All available nasopharyngeal swabs in M4RT buffer (*n* = 767) were tested using molecular tests. All available serum samples, after excluding missing visits or samples and missed or delayed tests (*n* = 754), were tested for RSV-pre- and -post-F binding and RSV-A2 neutralizing IgG antibody assays (**Figure 1A**).

### 2.3 Molecular tests and antibody measurements

Molecular testing: nasopharyngeal samples were tested for RSV and Influenza using the Xpert^®^ Xpress Flu/RSV assay by Cepheid; afterwards, the results were verified by RT-PCR developed by Glaxo Smith Kline (GSK). The detection limits were 304 copies/mL for RSV-A and 475 copies/mL for RSV-B. Viral load was quantified as RSV RNA copies per sample from 767 nasopharyngeal swabs in M4RT buffer.

Serum and mucosal antibody assays: RSV-pre-F and -post-F binding IgG antibody ELISA assays were performed by Janssen Pharmaceutical (13) and RSV-A2 µPRNT50 assays were developed and performed by Sanofi. Together with RSV molecular assays (point-of-care PCR and qPCR), the methods of the RSV-pre-F and -post-F binding IgG and RSV-A2 neutralizing IgG ELISAs were also described in detail elsewhere (5). For subset biomarker analyses, we randomly selected a subset of controls for RSV-G IgG and mucosal RSV-pre-F IgA/IgG ELISA, measured using methods developed by Sanofi.

A detailed description of the assay methodology can be found in the **Supplementary Text**.

### 2.4 Definition of infection group based on molecular and serology assays

First, participants were categorized into three infection groups based on molecular test results and respiratory infection symptoms during the RSV season. Participants with ARTI symptoms and positive molecular test (POCT PCR or qPCR) results for RSV were classified as RSV ARTI. Participants with ARTI symptoms but negative RSV molecular test results were classified as non-RSV ARTI. Participants who did not report ARTI symptoms and therefore were not tested were classified as no ARTI.

Second, participants were classified into three RSV infection groups based on both molecular and serology tests. Participants who exhibited ARTI symptoms and tested positive for RSV using the molecular tests were classified as RSV ARTI. By developing a study-specific algorithm, receiver operating characteristic (ROC) analysis, a serological threshold was associated with an RSV exposure per type of serum antibody titers. These thresholds were then used to identify participants with asymptomatic RSV from the previously defined no-ARTI group. Participants who did not report any ARTI symptoms over the season but exhibited any antibody fold change (FC) above the identified FC thresholds were classified as asymptomatic RSV ARTI. Participants who did not report ARTI symptoms over the season and exhibited FC lower than the identified FC thresholds were classified as no ARTI. We selected participants with asymptomatic RSV infection without ARTI symptoms to avoid false-negative RSV infections that may occur via molecular tests.

RSV disease severity was not assessed as there were no participants with severe RSV infection (hospitalization) identified in this cohort.

### 2.5 Statistical analysis

All data analyses and graphs were produced using the R software (14). Statistical difference between the groups in clinical and the demographic data were analyzed with the Kruskal–Wallis test for continuous data and the chi-squared test for categorical data. Raw antibody levels were log_2_-transformed. The results were expressed as median [min, max]. *t*-test was performed to compare baseline antibody levels. Logistic regression was used to relate binary variables to continuous variables. Odds ratio (OR) and 95% confidence interval (95% CI) were calculated using the glm package in R. ROC was calculated with the ROCR R package (15). No imputation was performed for the missing data. Complete case analyses were performed in the downstream analyses.

## 3 Results

### 3.1 Clinical and demographic characteristics of the cohort

Participants with missing serum samples or serology measurements and participants with missing or delayed (>7 days after the symptom onset) tested infections were excluded from the analysis. The remaining 754 participants were classified into three infection groups—RSV ARTI ( N=30 ), non-RSV ARTI ( N=386 ), and no ARTI ( N=338 ) (**Figure 1B**; **Table 1**)—to identify antibody-mediated protection of serum and mucosal antibodies by contrasting pre-season antibody titers of participants with symptomatic RSV ARTI and participants without RSV ARTI. Clinical and demographic characteristics of the three infection groups are described in **Table 1**. No difference in the number of RSV ARTI, non-RSV ARTI, and no-ARTI groups was observed for age, sex, ethnicity, comorbidities (long-term medical condition and cardiac or pulmonary disease), smoking, and having allergy. None of the participants in the RSV ARTI group were diabetic and had a lower BMI compared with the control groups. However, there might be a selection bias in these observations as two subjects with diabetes were excluded because of missing sample/data; hence, no difference was reported between the groups in the respective epidemiological study (5).

**Table 1:** Clinical and demographic characteristics of the cohort.

	No ARTI	Non-RSV ARTI	RSV ARTI	*p*-value
	(*N* = 338)	(*N* = 386)	(*N* = 30)	
Age 75+	186 (55.0%)	200 (51.8%)	18 (60.0%)	ns
Median [min, max]	76.0 [60.0, 95.0]	75.0 [60.0, 94.0]	75.5 [64.0, 89.0]	ns
Female gender	173 (51.2%)	212 (54.9%)	16 (53.3%)	ns
BMI median [min, max]	25.2 [17.6, 62.3]	25.7 [17.3, 61.0]	23.9 [17.1, 32.4]	<0.05*
Missing	4 (1.2%)	1 (0.3%)	0 (0%)	
Ethnicity Northwest European	327 (96.7%)	373 (96.6%)	27 (90.0%)	ns
Other	11 (3.3%)	11 (2.9%)	2 (6.7%)	
Missing	0 (0%)	2 (0.5%)	1 (3.3%)	
Long-term medical condition	207 (61.2%)	266 (68.9%)	20 (66.7%)	ns
Missing	4 (1.2%)	0 (0%)	0 (0%)	
Cardiac disease	62 (18.3%)	75 (19.4%)	6 (20.0%)	ns
Missing	4 (1.2%)	0 (0%)	0 (0%)	
Pulmonary disease	32 (9.5%)	43 (11.1%)	4 (13.3%)	ns
Missing	4 (1.2%)	0 (0%)	0 (0%)	
Any types of diabetes	13 (3.8%)	37 (9.6%)	0 (0%)	<0.05*
Missing	4 (1.2%)	0 (0%)	0 (0%)	
Smoking	31 (9.2%)	19 (4.9%)	3 (10.0%)	ns
Stopped	122 (36.1%)	166 (43.0%)	12 (40.0%)	
Missing	1 (0.3%)	0 (0%)	0 (0%)	
Allergy	79 (23.4%)	114 (29.5%)	8 (26.7%)	ns
Missing	6 (1.8%)	10 (2.6%)	1 (3.3%)	

Values are reported as median [minimum, maximum], number of participants, and (percentage %).

ARTI, acute respiratory tract infection; N, number of participants; ns, not significant; RSV, respiratory syncytial virus; *p-value below 0.05. p-values for Kruskal–Wallis test and chi-squared test for comparison of three groups are shown.

The second objective was to identify participants with asymptomatic RSV infection. Using thresholds generated from ROC analyses, the non-symptomatic RSV group was further divided into asymptomatic RSV ( N=43 ) and no ARTI ( N=295 ) (**Figure 1B**).

### 3.2 Antibody-mediated protection from RSV ARTI

Antibody levels at pre-RSV-season (baseline), RSV ARTI visit, and end-of-RSV-season visit are summarized in **Supplementary Table 1**. Sera IgG antibody levels between the RSV ARTI visit and pre-RSV-season visit were comparable, except for three participants showing a more than twofold increase in serum antibody titers at RSV visit compared with the preseason visit.

**Figure 2:**
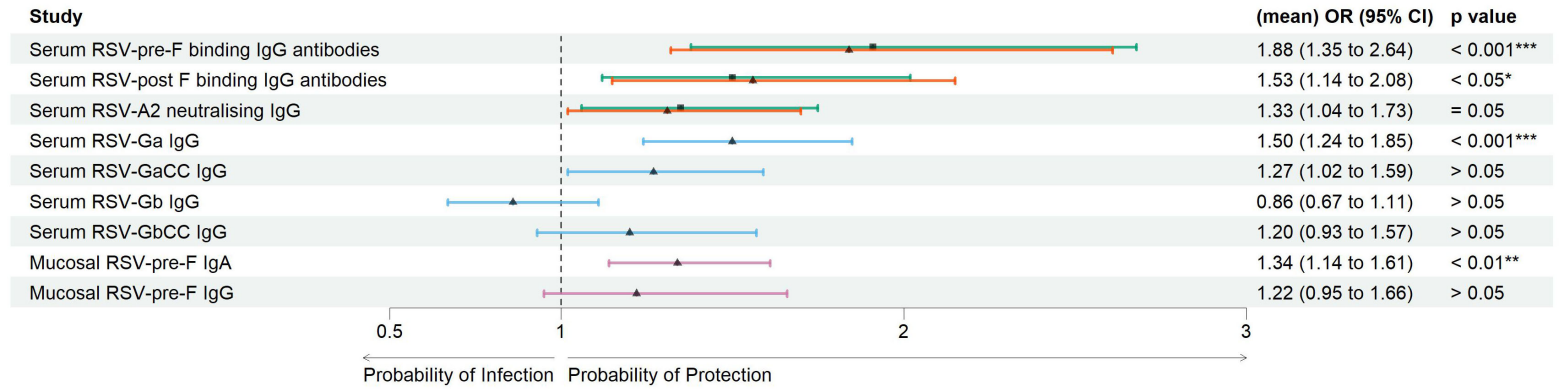
Correlates of protection of serum and mucosal antibodies. Forest plots showing the result of logistic regression analysis for the association of serum and mucosal antibodies with the probability of protection, or conversely symptomatic ARTI due to RSV. Arrows were colored for the following comparison groups. Green with square symbol for no ARTI (*N* = 338) versus RSV ARTI (*N* = 30), orange with triangle symbol for non-RSV (other) ARTI (*N* = 386) versus RSV ARTI (*N* = 30) in serum RSV-pre-F and -post-F binding IgG antibodies, blue with triangle symbol for a mixed group of controls (*N* = 119) versus RSV ARTI (*N* = 30) for the test in serum G binding IgG antibodies, and pink with triangle symbol for a subset group of controls (*N* = 45) versus RSV ARTI (*N* = 25) for the mucosal antibodies. Mean odds ratios (ORs) are reported for serum RSV-pre-F and -post-F binding IgG and neutralizing antibodies. Square or triangle dots for comparison of no ARTI versus RSV ARTI and other or subset group of other non-RSV ARTI versus RSV ARTI, respectively, represent the odds ratio, and the bars correspond to the confidence intervals (CIs) from 5% to 95%. Odds ratios and *p*-values were computed using the Fisher test described in the R glm package. *** represents a *p*-value < 0.001, ** represents a *p*-value < 0.01, and * represents a *p*-value < 0.05. ARTI, acute respiratory tract infection.

*Serum antibody-mediated protection:* Pre-season visit titers of RSV-pre-F and -post-F binding IgG antibodies were significantly lower ( p < 0.001 ) in the RSV ARTI group compared with the no-ARTI group with the following log_2_ median values, respectively: for RSV-pre-F binding IgG antibodies, 7.92 versus 8.55, and for RSV-post-F binding IgG antibodies, 7.68 versus 8.16 (**Supplementary Table 2**). The pre-RSV-season visit titers of RSV-A2 neutralizing IgG antibodies were significantly lower ( p < 0.05 ) in the RSV-ARTI group compared with the no-ARTI group with median log_2_ values 8.56 versus 9.28. To predict the probability of protection from RSV infection, logistic regression analyses were performed by comparing pre-RSV-season serum titers in the RSV ARTI group and the control groups (i.e., no ARTI and non-RSV ARTI) (**Figure 2**; **Supplementary Table 2**). RSV-pre-F binding IgG antibodies showed the strongest antibody-mediated protection from the RSV disease [no ARTI versus RSV ARTI; OR (95% CI): 1.91 (1.38–2.68)].

Pre-RSV-season visit log_2_ titers of serum RSV-Ga (RSV G ectodomain from subtype A) binding IgG antibodies were significantly lower ( p<0.001 ) in the RSV-ARTI group ( N=30 ) compared with the subset of controls ( N=119 ) with log_2_ median values: 10.6 versus 11.4 (**Supplementary Table 2**). Higher serum RSV Ga binding IgG antibody titers at the pre-RSV-season visit were significantly associated with protection from RSV ARTI with the following OR (95% CI): 1.50 (1.24-184); *p* < 0.001. No significant differences were observed ( p >0.05 ) for RSV-GaCC (peptide corresponding to the central conserved domain of RSV G subtype A), Gb (RSV G ectodomain from subtype B), and GbCC (peptide corresponding to the central conserved domain of RSV G subtype B) binding IgG antibodies (**Figure 2**; **Supplementary Table 2**).

*Mucosal antibody-mediated protection:* Pre-RSV-season visit log_2_ levels of RSV-pre-F binding IgA antibodies in the nasal mucosa were significantly lower ( p <0.01 ) in the RSV-ARTI group ( N=25 ) compared with the subset of controls ( N=45 ) with log_2_ median values: 3.58 versus 5.09. (**Supplementary Table 2**). Higher titers at pre-RSV-season visit were significantly associated with protection from RSV ARTI [OR (95% CI): 1.34 (1.14–1.61); p < 0.01 (**Figure 2**; **Supplementary Table 2**)]. In contrast, no significant association was observed for the pre-existing mucosal pre-F-specific IgG antibodies ( p = 0.22 ).

*Correlation of antibody measurements:* Log_2_ FC over the season of RSV-pre-F and -post-F binding IgG antibodies are strongly correlated (*r* = 0.90, *p* < 0.001). On the other hand, the correlation was lower between FC of RSV-pre-F and -post-F binding IgG antibodies and neutralizing IgG antibodies (*r* = 0.60 and *r* = 0.57, respectively, *p* < 0.001). The difference could be attributed to the nature of the assays (total antibody versus functional antibody measurement) and laboratory-related differences (test in different laboratories).

There was no or poor correlation between the FCs in mucosal pre-F binding IgA and IgG, RSV-pre-F binding (*r* = −0.03, *r* = −0.06, respectively, *p* > 0.05), or neutralizing IgG antibodies (*r* = 0.22, *r* = 0.35, respectively, *p* > 0.05), implying a lack of relationship between mucosal and serum antibodies, as described previously (11). RSV-Gb binding IgG antibodies showed a partial correlation with pre-F binding IgG antibodies (*r* = 0.61), which may be due to the high prevalence of serotype RSV-B infections among the participants.

### 3.3 Identification of asymptomatic RSV patients

**Figure 3:**
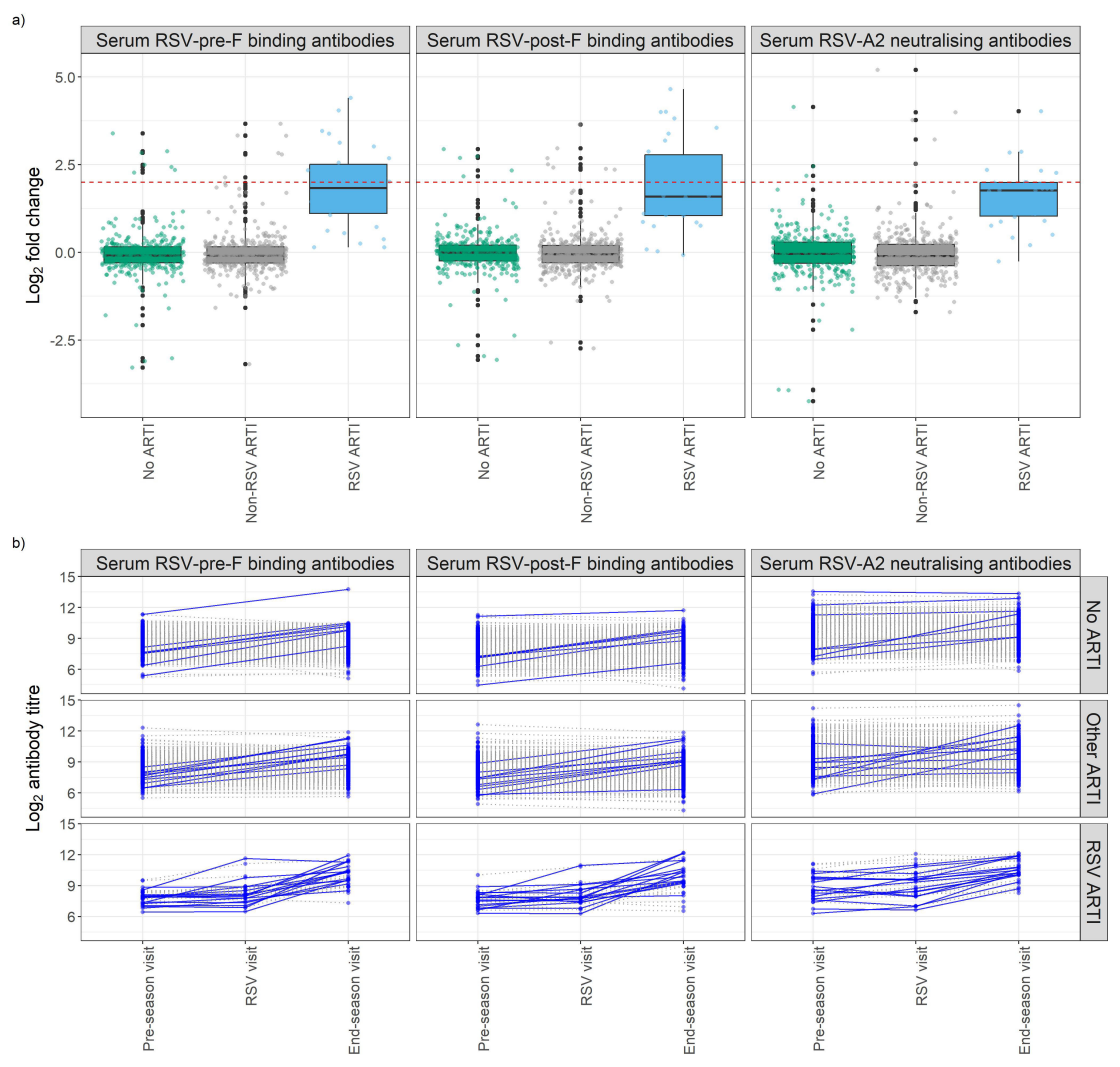
Antibody fold change (FC) over the season of study participants stratified by respiratory infection groups. **(A)** Box plots show the FC of RSV-pre-F and -post-F binding IgG and RSV-A2 neutralizing antibodies. The *Y*-axis shows log2 fold change of antibodies of participants stratified by their infection group based on molecular tests (POCT PCR and/or qPCR) and participants’ symptoms diary. Red dashed line shows log2 4 FC (=2). **(B)** Longitudinal analysis of RSV-pre-F and -post-F binding IgG antibodies and RSV-A2 neutralizing IgG antibody titers at pre-RSV-season, RSV, and end-of-season visits, stratified by respiratory infection status based on molecular tests and participants’ symptoms diary. Seroconversion is defined as having any antibody titer FC over the season above four. Participants with a positive seroconversion in any of the antibody titers were shown in dark blue and solid line. Participants with a negative seroconversion in any of the antibody titers were shown in gray and dashed line. Fold change (FC): ratio of end-of-season versus pre-RSV-season visit antibody levels. ARTI, acute respiratory tract infection; POCT, point-of-care test; PCR, polymerase chain reaction.

**Figure 4:**
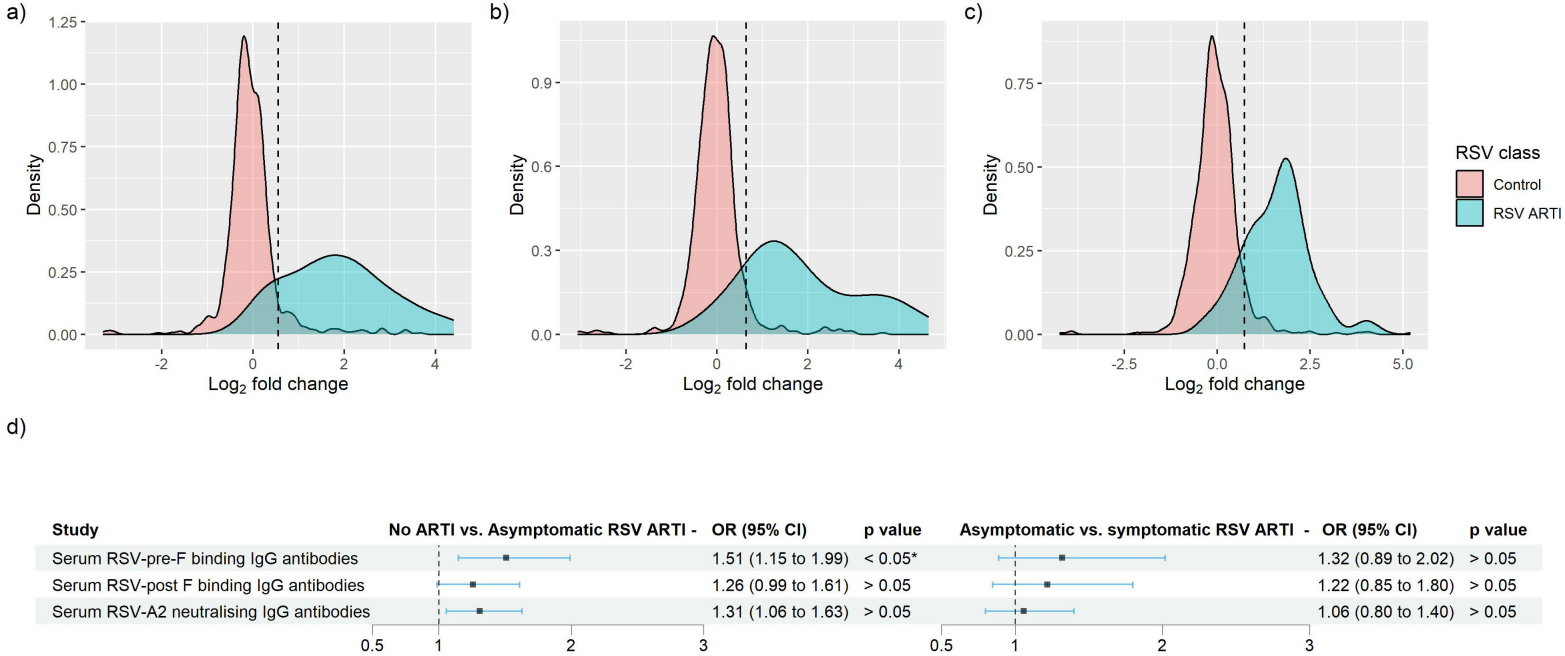
Identification of RSV asymptomatic participants through the receiver operating characteristic (ROC) curve method for RSV-pre-F and -post-F binding IgG, and RSV-A2 neutralizing Ig antibodies. **(A–C)** Log2-transformed FC of RSV ARTI (in blue) and controls were mapped. Black vertical dashed line shows the newly identified threshold for **(A)** RSV-pre-F binding IgG, **(B)** RSV-post-F binding IgG, and **(C)** RSV-A2 neutralizing IgG antibody assay. **(D)** Forest plots showing the result of logistic regression analysis for the association of serum antibodies with the probability of protection or infection for asymptomatic RSV. Square dots represent the odds ratio, and the bars correspond to the confidence intervals (CI) from 5% to 95%. Odds ratios and *p*-values were computed using the Fisher test described in the R glm package. * Represents a *p*-value < 0.05.

Overall, in the RSV ARTI group, median FC of RSV-pre-F and -post-F binding IgG and neutralizing antibodies were 3.57, 3.00, and 3.40, respectively (**Figure 3A**; **Supplementary Table 1**). A total of 16 (53.3%) participants had a greater than 4 FC in any of the tested antibody levels during the season; 14 (46.7%) did not pass the 4-FC threshold (**Figure 3B**). We performed an exploratory analysis to identify the best threshold using a data-driven method (i.e., ROC). ROC analysis identified RSV asymptomatic participants (false-positive rate < 0.1 and true-positive rate > 0.8) if their FC was higher than 1.4 FC for RSV-pre-F binding IgG (**Figure 4A**), 1.5 FC for RSV-post-F binding IgG (**Figure 4B**), and 1.6 FC for neutralizing IgG antibodies (**Figure 4C**). We deemed RSV asymptomatic (*N* = 43) any participant with no reported ARTI episode over the season and at least one antibody measurement (i.e., serum RSV-pre-F, RSV-post-F, or neutralizing IgG antibodies) above the identified FC threshold over the season. Time elapsed between infection and end-of-visit sampling did not correlate with the variance of FC over the season (**Supplementary Figures 1A–C**), while lower baseline RSV-pre-F binding and neutralizing IgG antibody levels correlated with higher fold increase (**Supplementary Figure 1D**).

Only RSV-pre-F binding IgG antibodies in RSV asymptomatic cases showed significantly lower levels of antibodies compared with the no-ARTI group ( p = 0.03 ) (**Supplementary Table 3**). Using logistic regression to predict the probability of protection from RSV asymptomatic infection, we found that both RSV-pre-F binding and RSV-A2 neutralizing IgG antibodies show antibody-mediated protection from asymptomatic RSV compared with the no-ARTI group, with the respective OR (95% CI) and *p*-values: 1.5 (1.14–1.99), p= 0.016,  and 1.3 (1.01 – 1.67), p = 0.033 (**Figure 4D**). There was no significant difference in the pre-RSV-season antibody levels between the asymptomatic infection and symptomatic infection, and no significant association for antibody-mediated protection from symptomatic RSV ARTI compared with asymptomatic RSV. Furthermore, we did not find significant differences between the ratios of RSV-pre- and post-F binding IgG antibodies of asymptomatic (1.18) and symptomatic RSV infection (1.16).

These results suggest that having higher levels of RSV-pre-F binding IgG antibodies may be associated with protection from RSV infections irrespective of the symptoms. A data-driven approach confirmed the protective role of higher baseline serum RSV-pre-F binding IgG antibodies.

## 4 Discussion

Our study is a secondary data analysis of a prospective cohort study conducted on RSV infection in community-dwelling older adults recruited prior to two consecutive RSV seasons and followed over one season, providing a snapshot of the health of older adults in three different European countries. Previous studies have focused on healthy or young adults (e.g., human challenge studies) or adults with comorbidities. However, age and comorbidities have been shown to affect RSV infection and severity (16). The RESCEU older adult study is an accurate representation of the population in which RSV infection is prevalent and may pose a health risk: older adults living in the community.

In infants, RSV-pre-F binding IgG antibodies have been proposed as an important biomarker for protection from the disease (17) and RSV-pre-F binding IgG antibodies were also associated with lower disease severity (18). Antibodies that bind to RSV-pre-F protein comprise most of the neutralizing antibody activity of the RSV-infected participants (19–21) and therefore offer unique possibilities for prevention from the RSV disease in older adults. Additionally, in a human challenge study in which healthy and young adults were recruited, the authors concluded that only mucosal pre-F binding IgA, but not IgG antibodies were protective against RSV infection (12). On the other hand, in a challenge model in which healthy younger and older adults were compared, serum IgGs correlated with protection and nasal IgA response was found to be impaired in older adults (22). Even though we observed an increase in nasal pre-F binding IgAs (log_2_-transformed RSV ARTI visit median 3.19, RSV convalescence median 4.67) and IgGs (log_2_-transformed RSV ARTI visit median 3.48, RSV convalescence median 4.52), our convalescence visit took place after 1–2 weeks of documented RSV ARTI (as opposed to 28th day in the cited study), which may explain the difference in results.

RSV-Ga is the RSV G from subtype A. The central domain of G is highly conserved between RSV subtypes and contains the CX3C motif, which is thought to interact with the CX3CR1 receptor on ciliated airway cells to initiate RSV infection (23–26). Monoclonal antibodies to the Gcc neutralize both RSV subtypes (24) while polyclonal responses to the entirety of the highly variable G ectodomain induced by natural infection are often subtype specific (27). To obtain a holistic view of G-directed antibody responses and their potential correlation with protection, both the full-length G ectodomain and the Gcc peptides corresponding to both RSV subtypes were utilized. Walsh et al. observed that both RSV-Ga and -Gb binding antibodies were higher in a control group and correlate with protection from RSV infection (9). In our study, we only concluded that RSV-Ga binding antibodies correlate with protection from RSV infection. This may be attributable to the difference in RSV subtype prevalence in different seasons and geographies.

There are some limitations in our study design. Because of the epidemiological nature of our study, convalescent sera were not available, which would have provided a better understanding on the kinetics of RSV antibody titers. The single post-infection sample collected at the end of the RSV season resulted in variations in time between infection and sample collection (2–7 months). Secondly, the control group labeled as “no ARTI” was likely composed of a mix of RSV-exposed and non-exposed individuals. Additional assays, such as mucosal anti-RSV G IgG and IgA antibody response, would have provided a more complete understanding of the serum and mucosal antibody-mediated protection in older adults. Finally, our focus was primarily on humoral immunity, neglecting other aspects of immunity such as antibody effector functions providing protection against RSV (28). Therefore, future studies into correlates of protection from RSV should include these factors to build towards a comprehensive model.

## 5 Conclusion

Our analyses show that RSV-pre-F and G IgG binding antibodies, along with mucosal RSV-pre-F binding IgA antibodies, may contribute to protection against RSV infection in community-dwelling older adults, and we demonstrate that higher levels of RSV-pre-F binding IgG antibodies are associated with protection from RSV infection regardless of symptoms. Furthermore, we highlight the potential protective role of RSV-G binding IgG antibodies, specifically the Ga and Gb subtypes, which warrant further investigation. Finally, we discuss the limitations of using a 4-FC threshold in antibody titers to detect asymptomatic RSV infections and propose a data-driven approach for their identification.

## Data Availability

The original contributions presented in the study are included in the article/**Supplementary Material**. Further inquiries can be directed to the corresponding author.

## RESCEU investigators

Harish Nair, Harry Campbell (University of Edinburgh), Philippe Beutels (Universiteit Antwerpen), LB (University Medical Center Utrecht), AP, Behnaz Ravanfar, Heather Rutter, Julie Allen (University of Oxford), Peter Openshaw (Imperial College London), Federico Martinon-Torres (Servicio Galego de Saude), Terho Heikkinen (University of Turku and Turku University Hospital), Adam Meijer (National Institute for Public Health and the Environment), Thea K. Fischer (Statens Serum Institut), Maarten van den Berge (University of Groningen), Carlo Giaquinto (PENTA Foundation), Michael Abram (AstraZeneca), Kena Swanson (Pfizer), Bishoy Rizkalla, Olivier Gruselle (GlaxoSmithKline), CV, Scott Gallichan, Vincent Pavot (Sanofi), JA (Janssen), Veena Kumar (Novavax), and Eva Molero (Team-It Research).
