# Supplementary material for: Serum and mucosal antibody-mediated protection and identification of asymptomatic respiratory syncytial virus infection in community-dwelling older adults in Europe
Source: Front Immunol. 2024 Oct 18;15:1448578. doi: 10.3389/fimmu.2024.1448578 (PMC11527605; doi:10.3389/fimmu.2024.1448578)
Supplement: Supplementary file 1 [file DataSheet1.pdf]

Supplementary Material

1 Supplementary Data

Molecular assays

Participants were followed-up during the RSV season by local study group. When the individual observed one or more respiratory symptoms (such as nasal congestion or discharge, cough, wheezing, or shortness of breath) for at least one day, a home visit was scheduled by a healthcare official to collect a nasopharyngeal sample for RSV and Influenza virus testing with a point-of-care test (POCT) by POCT, Xpert® Xpress Flu/RSV assay, Cepheid, Sunnyvale, CA, USA. The results were then verified with a quantitative reverse transcription-polymerase chain reaction (RT-PCR) assay for RSV-A and RSV-B with all available nasopharyngeal swabs collected in M4RT buffer (n=767). The RSV-A/B qRT-PCR protocol which targets the N gene was developed in Glaxo Smith Kline (GSK). The limit of detection for RSV-A was 304 copies/mL and for RSV-B was 475 copies/mL of swab. The RSV viral load is quantified as RSV RNA copies per sample.

RSV-pre-F and post-F ELISA

All available serum samples at pre-RSV-season, RSV infection, and post-season were run for RSV-pre-F and post-F binding antibody assay. RSV-pre-F and post-F binding antibody ELISA assays were performed with the method developed in Janssen Pharmaceutica[13]. In brief, RSV-pre-F and post-F coated streptavidin ELISA plates containing clinical samples and reference standards were incubated for 2 hours. HRP-conjugated mouse anti-human IgG (Fc) detection antibody were added. Antigen-specific IgG binding is measured with a luminescence readout. Anti-RSV-pre-F and anti-RSV-post-F concentrations were measured based on the standard curve by Gen5 software using the duplicate measurements. The antibody titres were reported in EU/L. For pre-F ELISA, the lower limit of quantification was 14.1 and the upper limit of quantification was 56224 EU/L. For RSV-post-F ELISA the lower limit of quantification was 6.6 EU/L and upper limit of quantification was 44280 EU/L.

Neutralising antibodies RSV-A2 µPRNT50

Neutralising antibodies RSV-A2 µPRNT50 assay was performed by a method developed by Sanofi using all available samples at pre-RSV-season, RSV visit, and end-of-season. Vero cells (ATCC CCL-81) were seeded at 30,000 cells/well in 96-well plates suitable for fluorescence reading one day prior infection. Serum samples were heat inactivated and 4-fold serially diluted from 1:20 to 1:81,920 in FluoroBrite DMEM supplemented with 1% Glutamax and 1% penicillin streptomycin (assay media). Diluted sera were combined 1:1 with RSV strain A2 expressing Green Florescent Protein reporter diluted in assay media supplemented with 10% guinea pig complement (Cedarlane Labs, CL5000) and incubated for one hour at room temperature. The serum-virus mixtures were then added to the cell plates and incubated for 24 hours at 37°C. The plates were then read on a high content imager and the florescent events were quantified. Serum 50% neutralising titres were calculated using 4-parameter logistics regression in SoftMax GxP software.

Mucosal antibodies

Mucosal RSV-pre-F- IgA and IgG antibodies were measured in a subset of nasopharyngeal samples using a method developed by Sanofi. To control for variability in mucosal sampling, the RSV-pre-F -IgA and IgG titres were normalized to the ng/Ml of total IgG in the samples. To quantify total IgG, ELISA plates were coated with 1 µg/Ml of Goat Anti-Human IgG (Jackson Immunoresearch, 109-005-098) and incubated overnight at 4°C. The plates were washed with PBS-Tween 0.1%, blocked with PBS-Tween 0.1%-BSA 3% for one hour at 37°C, then washed 6 times. A serial dilution series of purified Human IgG standard (Alpha Diagnostic International 20007-1-1) was added to each plate, with serial dilution series of the samples added to the remainder of the plate. The plates were incubated for one hour at 37°C then washed 6 times. Biotin conjugated anti-human IgG (Jackson Immunoresearch, 109-065-088) was added for one hour at 37°C, the plates were washed 6 times, then incubated with DELFIA® Europium-labelled streptavidin for one hour at 37°C. Plates were then washed 6 times and developed with DELFIA® Enhancement Solution for 20 minutes at room temperature with gentle rocking. Europium time-resolved fluorescence was determined, the Total IgG in the samples calculated by interpolating onto the standard curve and reported as ng/Ml.

To quantify RSV-pre-F - IgA and IgG, alternating rows of ELISA plates were coated with 1 µg/Ml of RSV F protein in DPBS or with DPBS alone. The coated plates were incubated overnight at 4°C and then washed with PBS-Tween 0.1% and blocked with PBS-Tween 0.1%-BSA 3% (Block) for one hour at 37°C, then washed. Samples were diluted 1:3 in block and added to adjacent, protein coated and mock coated, wells, and incubated for one hour at 37°C. Plates were washed 6 times, then incubated with Biotin conjugated anti-human IgG (Jackson Immunoresearch, 109-065-088) or IgA (Jackson Immunoresearch, 109-065-011) for one hour at 37°C. Plates were washed 6 times, then incubated with DELFIA® Europium-labelled streptavidin for one hour at 37°C. Plates were then washed 6 times and developed with DELFIA® Enhancement Solution for 20 minutes at room temperature with gentle rocking. Europium time-resolved fluorescence was determined, the background signal in the DPBS coated wells was subtracted from the corresponding protein coated wells and this quantity was then multiplied by the dilution factor. The results were reported as Relative Florescent Units (RFU) divided by Total IgG signal.

G-ELISA antibodies

G binding antibody ELISA assays were quantified in a subset of sera samples with the method developed by Sanofi. Briefly, ELISA plates were coated with 1 µg/Ml of either recombinant G protein from RSV subgroup A or B, or peptides corresponding to the central conserved domains these proteins. The coated plates were incubated overnight at 4°C, then blocked with PBS-Tween 0.05%-BSA 1% for one hour. Sera were three-fold serially diluted in PBS-Tween 0.05%-BSA 1% in the coated plates. After a one-hour incubation at Room Temperature (RT) plates were washed with PBS-Tween 0.1% and incubated for one hour at RT with a donkey anti-mouse IgG-HRP (Jackson Immunoresearch, cat. 715-035-151). Plates were then washed and developed with TMB substrate. The colorimetric reaction was stopped with TMB Stop Solution, Optical Densities (OD) were measured at 450 nm and titres quantified through interpolating the serum dilutions at OD 0.2 via 4-parameter logistic regression.

The G proteins are catalog items from SinoBiological (Ga = 11070-V08H, Gb 13029-V08H). GaCC amino acid sequence is RQNKPPNKPNNDFHFEVFNFVPCSICSNNPTCWAICKRIP and that of GbCC is RKNPPKKPKDDYHFEVFNFVPCSICGNNQLCKSICKTIP. The

peptides were synthesized using standard solid phase synthesis, cleavage & purification conditions. Orthogonal chemistry was used to avoid ambiguity in the configuration of the disulfide bonds. Proper disulfide pairing was confirmed using disulfide mapping by LC-MS/MS. Extensive assessment of mAb binding was performed and is the subject of a manuscript in preparation.

1.1 Supplementary Figures

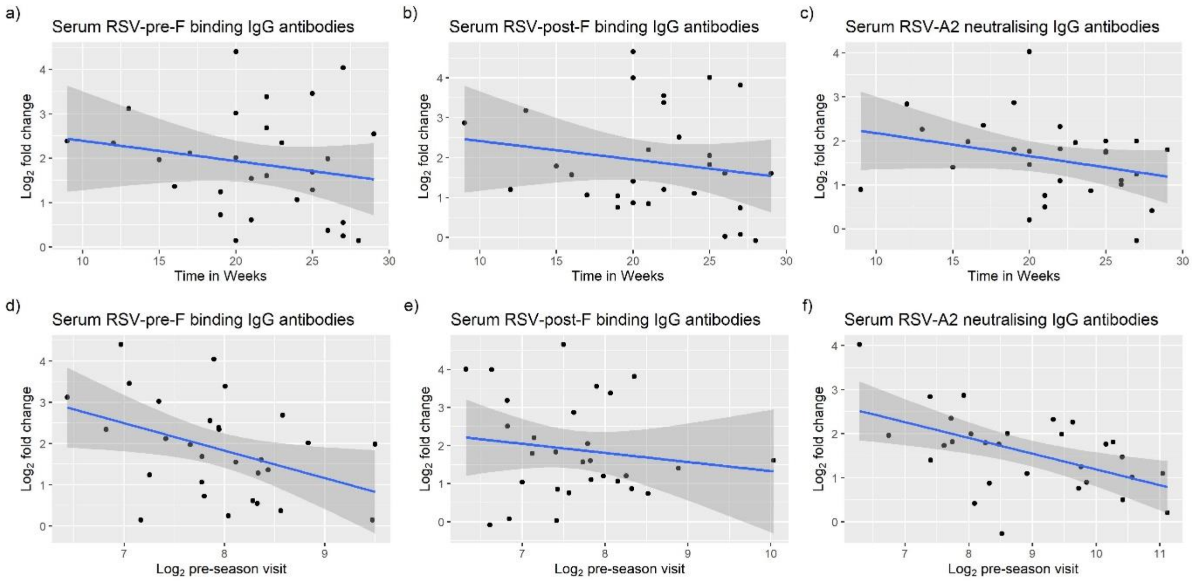

Supplementary Figure S1: Effect of time between RSV ARTI and end-of-season visit infection and baseline antibody levels on fold change (FC) of antibodies over the season.

a-c) Y-axis shows log2 FC and x-axis shows time between RSV ARTI and end-of-season visit infection in weeks. The pearson correlation is estimated for a) serum RSV-pre-F binding IgG antibodies (-0.20, p value > 0.05), b) serum RSV-post-F binding IgG antibodies (-0.18, p value > 0.05), and c) serum RSV-A2 neutralising IgG antibodies (-0.29, p value > 0.05). d-f) Y-axis shows log2 FC and x-axis shows log2 antibody levels at pre-season visit. The pearson correlation is estimated for d) serum RSV-pre-F binding IgG antibodies (-0.41, p value = 0.03), e) serum RSV-post-F binding IgG antibodies (-0.14, p value > 0.05), and f) serum RSV-A2 neutralising IgG antibodies (-0.52, p value = 0.003). Linear model is applied, and each dot represents data point of one subject.

1.2 Supplementary Tables

Supplementary Table 1: Descriptive results of log_2 transformed antibody levels at pre-RSV-season, RSV ARTI, and end-of-season visit for each comparison groups

Group	Statistics	Pre-RSV-season	RSV ARTI	End-of-RSV-season	FC over the season
**Serum RSV-pre-F binding IgG (Log$_2$ transformed)**					
No ARTI	Mean (SD)	8.54 (0.996)	na	8.50 (1.06)	1.09 (0.887)
	Median [Min, Max]	8.55 [5.27, 11.3]	na	8.51 [5.14, 13.8]	0.938 [0.102, 10.5]
Non-RSV ARTI	Mean (SD)	8.48 (1.00)	na	8.46 (1.01)	1.13 (1.08)
	Median [Min, Max]	8.44 [5.52, 12.3]	na	8.43 [5.64, 11.9]	0.935 [0.110, 12.7]
RSV ARTI	Mean (SD)	7.92 (0.706)	8.35 (1.16)	9.78 (1.08)	5.01 (4.64)
	Median [Min, Max]	7.92 [6.43, 9.50]	8.17 [6.48, 11.6]	9.62 [7.31, 11.9]	3.57 [1.10, 21.1]
	Missing	na	1 (3.3%)	na	
**Serum RSV-post-F binding IgG (Log$_2$ transformed)**					
No ARTI	Mean (SD)	8.13 (1.10)	na	8.12 (1.13)	1.09 (0.699)
	Median [Min, Max]	8.16 [4.49, 11.3]	na	8.19 [4.14, 11.7]	0.991 [0.119, 7.69]
Non-RSV ARTI	Mean (SD)	8.14 (1.10)	na	8.13 (1.12)	1.11 (0.916)
	Median [Min, Max]	8.03 [4.92, 12.6]	na	8.07 [4.30, 11.8]	0.962 [0.150, 12.5]
RSV ARTI	Mean (SD)	7.65 (0.767)	8.05 (1.08)	9.52 (1.40)	5.53 (5.82)
	Median [Min, Max]	7.68 [6.32, 10.0]	7.79 [6.28, 10.9]	9.32 [6.52, 12.2]	3.00 [0.946, 25.2]
	Missing	na	1 (3.3%)	na	
**Serum RSV-A2 neutralising antibodies (Log$_2$ transformed)**					
No ARTI	Mean (SD)	9.38 (1.32)	na	9.34 (1.29)	1.11 (1.03)
	Median [Min, Max]	9.28 [5.55, 13.6]	na	9.34 [5.86, 13.4]	0.967 [0.0528, 17.7]
Non-RSV ARTI	Mean (SD)	9.31 (1.31)	na	9.27 (1.33)	1.20 (2.16)
	Median [Min, Max]	9.24 [5.83, 14.2]	na	9.15 [6.13, 14.5]	0.928 [0.307, 36.8]
RSV ARTI	Mean (SD)	8.87 (1.28)	9.21 (1.47)	9.34 (1.29)	3.66 (2.85)

	Median [Min, Max]	8.56 [6.29, 11.1]	9.35 [6.63, 12.1]	9.34 [5.86, 13.4]	3.40 [0.834, 16.3]
	Missing	na	1 (3.3%)	na	

ARTI: acute respiratory tract infection, FC: fold change (ratio of end-of-RSV-season versus pre-RSV-season visit antibody levels). na= not available, SD= standard deviation.

Supplementary Table 2: Raw data of the antibody levels of RSV–specific antibodies with probability of protection from RSV ARTI.

	**No ARTI versus RSV ARTI**				
	**No ARTI (N=338)**	**RSV ARTI (N=30)**	**t-test**	**Logistic regression**	
	median [min-max]	median [min-max]	P value	OR [5-95%]	P value
Serum RSV-pre-F binding IgG antibodies	8.55 [5.27, 11.3]	7.92 [6.43, 9.50]	< 0.001 ***	1.91 [1.38 – 2.68]	0.001 **
Serum RSV-post-F binding IgG antibodies	8.16 [4.49, 11.3]	7.68 [6.32, 10.0]	< 0.001 ***	1.5 [1.12 – 2.02]	0.02 *
Serum RSV-A2 neutralising IgG antibodies	9.28 [5.55, 13.6]	8.56 [6.29, 11.1]	0.05 *	1.35 [1.06 – 1.75]	0.05 *
	**Non-RSV ARTI versus RSV ARTI**				
	**Non-RSV ARTI (N=386)**	**RSV ARTI (N=30)**	**t-test**	**Logistic regression**	
	median [min-max]	median [min-max]	P value	OR [5-95%]	P value
Serum RSV-pre-F binding IgG antibodies	8.44 [5.52, 12.3]	7.92 [6.43, 9.50]	< 0.001 ***	1.84 [1.32 – 2.61]	< 0.01 **
Serum RSV-post-F binding IgG antibodies	8.03 [4.92, 12.6]	7.68 [6.32, 10.0]	< 0.01 **	1.56 [1.15 – 2.15]	0.02 *
Serum RSV-A2 neutralising IgG antibodies	9.24 [5.83, 14.2]	8.56 [6.29, 11.1]	0.10 (n.s.)	1.31 [1.02 – 1.70]	0.08 (n.s.)
	**Controls versus RSV ARTI**				
	**Controls (N=119)**	**RSV ARTI (N=30)**	**t-test**	**Logistic regression**	
	median [min-max]	median [min-max]	P value	OR [5-95%]	P value
Serum RSV-Ga IgG antibodies	11.4 [8.23, 16.2]	10.6 [6.64, 13.0]	< 0.001 ***	1.50 [1.24 – 1.84]	< 0.001 ***

Serum RSV-GaCC IgG antibodies	11.4 [8.23, 16.2]	11.4 [8.23, 14.6]	0.08 (n.s.)	1.27 [1.02 – 1.59]	0.08 (n.s.)
Serum RSV-Gb IgG antibodies	11.4 [8.23, 14.6]	11.4 [8.23, 14.6]	0.35 (n.s.)	0.86 [0.67 – 1.11]	0.35 (n.s.)
Serum RSV-GbCC IgG antibodies	9.81 [6.64, 13.0]	9.81 [6.64, 13.0]	0.26 (n.s.)	1.20 [0.93 – 1.57]	0.25 (n.s.)
	**Controls versus RSV ARTI**				
	**Controls (N=45)**	**RSV ARTI (N=25)**	**t-test**	**Logistic regression**	
	median [min-max]	median [min-max]	P value	OR [5-95%]	P value
Mucosal RSV-pre-F binding IgA antibodies	5.09 [-0.04, 9.80]	3.58 [-3.73, 6.75]	< 0.01 **	1.34 [1.14 – 1.61]	< 0.01 **
Mucosal RSV-pre-F binding IgG antibodies	4.18 [-5.24, 7.90]	3.58 [1.12, 5.26]	0.14 (n.s.)	1.22 [0.95 – 1.66]	0.22 (n.s.)

Log_2 median [minimum, maximum] were shown. T-test is performed to compare RSV-infected participants versus non-infected controls (no ARTI, non-RSV ARTI, and mixed "controls"). Logistic regression model is used to estimate the correlation for protection from RSV ARTI disease. *** represents a p value 0.001, ** represents a p value < 0.01, and * represents a p value < 0.05, n.s. represents not significant with a p value > 0.05. ARTI: acute respiratory tract infection.

Supplementary Table 3: Raw data of the antibody levels of RSV–specific antibodies with probability of protection from asymptomatic RSV infection.

	**No ARTI (N=295)**	**Asympto-matic RSV (N=43)**		**Logistic regression**	
	median [min-max]	median [min-max]	P value	OR [5-95%]	P value
Serum RSV-pre-F binding antibodies	8.57 [5.50, 11.3]	8.26 [5.27, 11.3]	0.03 *	1.51 [1.15 – 1.99]	0.01 *
Serum RSV-post-F binding antibodies	8.19 [5.34, 11.3]	7.93 [4.49, 11.2]	0.17 (n.s.)	1.26 [0.99 – 1.61]	0.12 (n.s.)
Serum RSV-A2 neutralising antibodies	9.36 [5.73, 13.2]	8.75 [5.55, 13.6]	0.08 (n.s.)	1.31 [1.06 – 1.63]	0.04 *
	**No ARTI (N=295)**	**RSV ARTI (N=30)**			
Serum RSV-pre-F binding antibodies	8.57 [5.50, 11.3]	7.92 [6.43, 9.50]	< 0.001 ***	2.21 [1.55-3.22]	< 0.001 ***
Serum RSV-post-F binding antibodies	8.19 [5.34, 11.3]	7.68 [6.32, 10.0]	0.002 **	1.61 [1.18 – 2.21]	0.013 *
Serum RSV-A2 neutralising antibodies	9.36 [5.73, 13.2]	8.56 [6.29, 11.1]	0.03 *	1.44 [1.11 – 1.88]	0.023 *
	**Asymptomatic RSV (N=43)**	**RSV ARTI (N=30)**			
Serum RSV-pre-F binding antibodies	8.26 [5.27, 11.3]	7.92 [6.43, 9.50]	0.23 (n.s.)	1.32 [0.89-2.02]	0.26 (n.s.)
Serum RSV-post-F binding antibodies	7.93 [4.49, 11.2]	7.68 [6.32, 10.0]	0.34 (n.s.)	1.22 [0.85 – 1.80]	0.37 (n.s.)
Serum RSV-A2 neutralising antibodies	8.75 [5.55, 13.6]	8.56 [6.29, 11.1]	0.74 (n.s.)	1.06 [0.80 – 1.40]	0.74 (n.s.)

Log_2 median [minimum, maximum] were shown. T-test is performed to compare asymptomatic RSV infected participants ($N = 43$) with symptomatic RSV ARTI ($N = 30$) and No ARTI controls ($N = 295$). Logistic regression model is used to estimate the correlation for protection from RSV ARTI disease. *** represents a p value < 0.001, ** represents a p value < 0.01, and * represents a p value < 0.05. ARTI: acute respiratory tract infection, n.s. represents not significant with a p value > 0.05.
